# Topological Avenue for Efficient Decontamination of Large Volumes of Fluids via UVC Irradiation of Packed Metamaterials

**DOI:** 10.3390/ma16134559

**Published:** 2023-06-24

**Authors:** Nicolae A. Enaki, Ion Munteanu, Tatiana Paslari, Marina Turcan, Elena Starodub, Sergiu Bazgan, Diana Podoleanu, Carmen Ristoscu, Sinziana Anghel, Maria Badiceanu, Ion N. Mihailescu

**Affiliations:** 1 Quantum Optics and Kinetic Processes Laboratory, Institute of Applied Physics, Moldova State University, 5 Academiei Str., MD2028 Chișinău, Moldova; enache_nicolae@yahoo.com (N.A.E.); goretz19@yahoo.com (I.M.); tatianapaslari@gmail.com (T.P.); tmaryna@yahoo.com (M.T.); nutza.starodub@gmail.com (E.S.); bizgan_s@yahoo.com (S.B.); podoleanud@mail.ru (D.P.); 2 Laser-Surface-Plasma Interactions Laboratory, National Institute for Lasers, Plasma and Radiation Physics (INFLPR), Magurele, Ilfov, RO-077125 Bucharest, Romania; carmen.ristoscu@inflpr.ro (C.R.); maria.badiceanu@gmail.com (M.B.); 3 Faculty of Physics, University of Bucharest, Magurele, Ilfov, RO-77125 Bucharest, Romania

**Keywords:** super-packed metamaterial structures, UVC irradiation, fluids decontamination, yeast/pathogen colonies

## Abstract

Nowadays, metamaterials application enjoys notoriety in fluid decontamination and pathogen annihilation, which are frequently present in polluted fluids (e.g., water, blood, blood plasma, air or other gases). The depollution effect is largely enhanced by UVC irradiation. The novelty of this contribution comes from the significant increase by packing of the total surface of metamaterials in contact with contaminated fluids. Packed metamaterial samples are subjected to UVC irradiation, with expected advantages for implant sterilization and long-term prevention of nosocomial infections over large clinical areas. The novel aspect of the investigation consists of a combination of big and small elements of the metamaterial to optimize the above effects connected with fluids and irradiation. The big elements allow the radiation to penetrate deep inside the fluid, and the small elements optimally disperse this radiation toward deeper regions of the metamaterial. A packing scheme of smaller, in-between large metamaterial spheres and fibres is proposed for promoting enhanced depollution against pathogen agents. It is demonstrated that the total surface of metamaterials in contact with contaminated fluids/surface is significantly increased as a result of packing. This opens, in our opinion, new auspicious perspectives in the construction of novel equipment with high sensibility in the detection and decontamination of microorganisms.

## 1. Introduction

One major concern in these pandemic times is the implementation of new opportunities to increase the efficiency of the decontamination rate of fluids. Thus, according to Refs. [1,2] the efficiency of UV irradiation methods for the inactivation of coronavirus could reach 90%. In order to minimize the equipment size, the same experiments were conducted with UVC (i.e., within 100–280 nm spectral range) LEDs [3] when the decontamination surface efficiency, the sources’ position (separation distance and exposure angle) was increased to 99.93% in the first 20 s.

Pathogen photoinactivation efficiency was investigated in Ref. [4] with respect to UVC dose, separation distance, and angular position on the surface. If the intensity of UVC radiation decreases, with increasing distance and angle of application, the use of fibre optics [5] becomes mandatory for directing the radiation towards the infected points or tissue.

Nowadays for life-threatening pandemics such as COVID-19, the development of novel, fast and cost-efficient techniques for virus detection [6,7] is required. The possibility of using hollow fibres fabricated from transparent materials within the UVC range through which fluids flow was investigated for this purpose [8,9,10,11,12].

For quick decontamination of large volumes, a new architecture of fluid channels between the metamaterial elements [13,14,15,16] obtained by the insertion of smaller elements of metamaterial in-between big ones was proposed. This approach opens the supplementary contact surfaces for UVC radiation with the fluids. The 3D channels for contamination fluids generated inside packed metamaterials drastically eliminate the friction between the liquid and metamaterial characteristics [8,9,10,11,12].

To improve the contact surface between the pathogens and UVC radiation, we used a combination of metamaterial elements such as entire or crushed spheres and fibres in packing arrangements. Our analyses are focused on the fabrication of a new class of equipment based on composite metamaterial structures, of nano- to micro-level, via exploiting the optical contact zone between designed metamaterial elements with various structural sizes. This combination of thin and thick elements of composite metamaterials may drastically improve the contact surface with contaminated object fluids/solid surfaces.

To treat surface infections within the region between the implant and cellular tissue, a new optical system is proposed. To boost the penetration of the UVC radiation into the fluids, we have tried to minimize the elements of quasi-periodical dispersion structures, which lead to an increase in the free contamination zone. This could be counteracted by the evanescent waves’ [17,18] penetration action, which evolves proportionally with the metamaterial elements’ density multiplied by their total surface. Along with this line, the best contact surface consists of drastically reduced, entire, or crushed spheres and optical fibres.

This concept does not nevertheless operate in the case of profound penetration depth of radiation inside contaminated fluids because of two basic phenomena. The first one is connected to the intense scattering of the UVC radiation on the surface of the packed metamaterial elements consisting of entire or crushed spheres and optical fibres of small diameter. The effective volume of metamaterial penetrated by contaminated fluid will not be therefore completely decontaminated. The second phenomenon is related to the increase in fluid resistance when in contact with metamaterial elements of smaller dimensions. Thus, experiments showed that the reflection increase at the contact between the elements of metamaterials becomes a major obstacle against the deeper penetration of radiation inside a metamaterial.

To overcome these apparent contradictions, we resort to composite metamaterials, consisting of a mix of big and small optical elements in contact with the contaminated fluid. The big elements allow UVC radiation to cross a large distance inside the contaminated liquids, while the small ones occupying the free space in between them are able to ensure the optimum dispersion of radiation in large volumes, as confirmed when using crushed quartz elements.

The work is organized as follows: a theoretical approach first describes the decontamination based on new composite metamaterials which consist of a mixture of big and small elements (spheres, optical fibres, and/or crushed elements). Experimental results were next introduced for quartz elements with different dimensions and geometries in the packing structures. The agreement between theoretical predictions and experimental results was confirmed based upon statistical bases.

## 2. Packing of Metamaterial Elements

The space in between metamaterial (e.g., quartz) elements, spheres, or fibers, can be filled up with smaller fragments, so that the radiation can be “captured” inside the tube in order to secure maximum antipathogen activity. Aspects of sphere packing including evanescent zones were mentioned in Refs. [17,18,19,20,21,22] while possibilities for modification of construction systems of connected fibers were discussed in references [23,24], but they refer to different research and application fields. Our strategy is based on the packing geometries of composite metamaterial elements. This approach is expected to promote a substantial enhancement of both UVC decontamination on the surface in contact with fluids as well as the decontamination rate. The task is to design and fabricate a new, compact decontaminator of fluids operating via optical contact between metamaterial elements with various dimensions. More precisely, the combination of thick and thin elements of composite metamaterials of either fragments or optical fibers is proposed.

### 2.1. Entire or Crushed Spheres

The free space in between big quartz spheres can be described by a filling-up factor, which is a function of the packing structure of metamaterials. The space can be described with respect to the atomic packing density, $V_{f}=V\left(1-\rho \right)$, where $V_{f}$ is the free volume between spheres, V is the total volume and ρ the cell density, which depends on the packing structure of the metamaterial.

**Figure 1:**
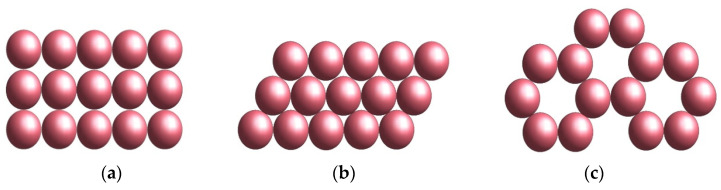
Planar packing of spheres in the case of: (**a**) Square packing; (**b**) Hexagonal packing; (**c**) Basic hexagonal structure with missing spheres.

**Figure 2:**
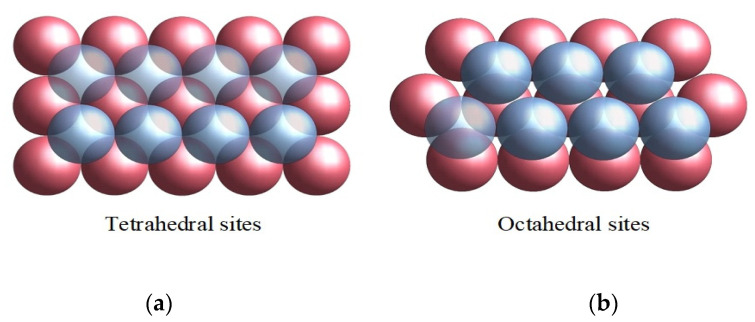
(**a**) Tetrahedral arrangements of quartz spheres in packing; (**b**) Octahedral lattice of quartz spheres in packing.

In the case of a hexagonal lattice arrangement, as shown in Figure 1b,c, the density is $\rho =\pi /3\sqrt{3}=0.6086$, which is superior to tetrahedral lattice packing (Figure 2), for which $\rho =\pi \sqrt{3}/16=0.3401$. This means that the free volume in the case of tetragonal packing is higher than in the hexagonal case.

Our option to analyse the decontamination efficiency of the contact surface between the metamaterials, as photonic crystals or fibres, was the use of a combination of metamaterial elements such as entire or crushed spheres and fibres in a packing arrangement, according to data in Ref. [13]. The fabrication of a suitable material is desired, to both ensure good penetration of UVC radiation into the metamaterial elements and easy propagation across contaminated fluids. One next calculates, on this basis, the effective decontamination volume, which is proportional to the contact surface between the contaminated fluid and metamaterials (entire spheres or sphere fragments) in the respective arrangement (packing).

It is generally accepted that the penetration depth, k, inside the free space in-between spheres, is directly proportional to the radiation wavelength and inversely proportional to the difference between the refractive indexes of metamaterial, $n_{m}$ and contaminated fluid $n_{f}$. κ can be written as: $\kappa \sim \lambda /\left[2\pi \sqrt{n_{m}^{2}-n_{f}^{2}}\right]$. It follows that the effective volume is $V_{u}=\kappa S$ where S stands for the contact surface of metamaterial with the contaminated fluid. This volume is inferior to the free volume in-between spheres, $V_{f}$. The relation between the sphere’s diameter, D, and the total surface of spheres reads as: $S=\pi D^{2}N$, where N stands for the total number of spheres. If one considers $L_{i}$, where $i=\left(x,y,z\right)$ a direction in space, the volume $V=L_{x}L_{y}L_{z}$ is filled up with, $N_{i}=L_{i}/D$ which is the number of spheres in each direction, cumulating a total number of spheres in the cell of $N=N_{x}N_{y}N_{z}$. This number is proportional to the volume: $N\sim V/D^{3}$. The effective decontamination volume around packed spheres is proportional to the penetration depth, κ~λ, and inversely proportional to the diameter of sphere, $V_{u}\sim V\lambda /D$. One notices that the free volume $V_{f}$ in-between spheres is $V_{fi}=V{\left(1-\rho \right)}^{\left(i-1\right)}$, in the case of smaller diameter spheres $D_{i-1}$.

**Figure 3:**
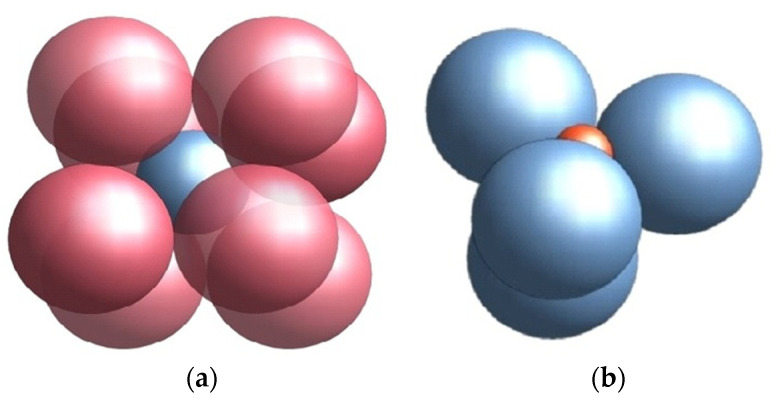
(**a**) Packing of small spheres in between big ones in a cubic arrangement; (**b**) Packing of small spheres into cells with tetragonal lattice.

The option to resort to small spheres is, however, not appropriate for the decontamination of fluids. On the one hand, the friction and resistance of the liquid in between grains increase with the reduction of dimensions. On the other hand, UVC radiation suffers multiple reflections onto contact surfaces between small spheres. We therefore propose a packing procedure with structures combining big and small spheres. In this respect, one should first identify the resonances between the gallery modes of the waves in the case of the two spheres of different diameters, in Figure 3 denoted $\left(a\right)$ and $\left(b\right)$, respectively.

**Figure 4:**
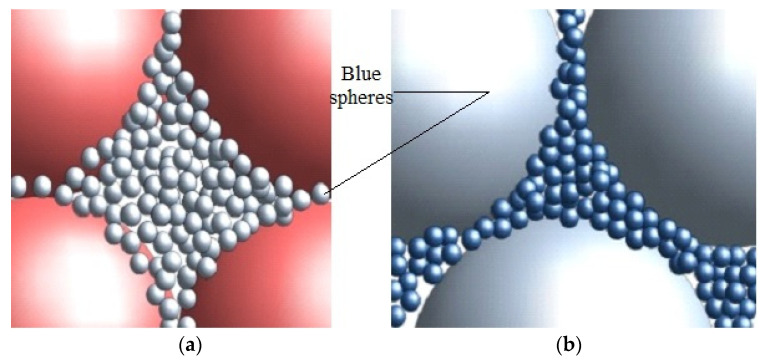
(**a**) Scheme of big spheres with a diameter d_1_ with a space in-between filled up with spheres of $D_{2}=K$, diameter; (**b**) Smaller spheres with a diameter $D_{3}=K^{2}$ filling up the space in-between the spheres of a diameter $D_{2}$. The free space filling could be continued till the packing of the smaller spheres reaches the diameter $D_{n}=K^{n-1}$.

In the case of spheres with a diameter of the order of $D_{3}=D_{1}\left(2-\sqrt{3}\right)$, the resonance is reached for practically any dimension of spheres (Figure 4).

It should be noted that the light-blue spheres in Figure 4a are in fact visible at a larger magnification 150× in Figure 4b showing that the small spheres (dark-blue) fill in the free spaces in between the large ones (light-blue) (Figure 4b). The hole assembly occupies the free spaces in between the big red spheres in Figure 4a.

As a result of the filling process, the free space in between the big red spheres diminishes while the volume of handled contaminated fluid is also decreased but the contact surface between fluid and evanescent zone is significantly increased.

The resonance in between micronic systems of different sizes becomes possible whenever the light wave is considered standing (inside its own quantum states). To further expand the contact surface, one may fill up the free space between smaller spheres. The total surface of $i{\mathrm{th}}^{}$-species is $S_{i}=4\pi V_{fi}/D_{i}$. Here, $V_{fi}=V{\left(1-\rho \right)}^{\left(i-1\right)}$ stands for the remanent free volume after inserting spheres with an ith diameter. The di diameter of *i*-type spheres is connected to one of the “i−1” spheres by the relation $D_{i}=K^{-1}D_{i}^{-1}$. Here, $K^{-1}$ is an order parameter less than unity. If one introduces a second type of spheres into the free space of a cubic lattice, the expression for the total surface is: $S=S_{1}+S_{2}$.

Here, $S_{1}=\pi D_{1}^{2}N$ and $S_{2}=\pi {\left(\sqrt{3}-1\right)}^{2}D_{1}^{2}$ are the surface of the big and small spheres placed in the two lattices with the same number of nodes, *N*, in optical contact. One notes that the small spheres with a diameter of $D_{2}=\left(\sqrt{3}-1\right)D_{1}$ are placed in the center of the cubes of the big one, but with no direct contact. In this case, $K=1/\left(\sqrt{3}-1\right)$. The total surface in contact with the contaminated fluid $S=\pi D_{1}^{2}N\left(5-2\sqrt{3}\right)$ increases with $0.56S_{1}$ with respect to the metamaterial consisting of the packed spheres of the same diameter.

One can continue to place smaller and smaller spheres inside the cubic structure. The spheres are introduced across each face of the cube while the packing starts from the centre of the cube. The distance between small spheres (with a diameter *D*_3_) and big spheres with a diameter *D*_1_ were estimated to be $D_{3}^{*}=D_{1}\left(\sqrt{2}-1\right)$. The distance $D_{3}^{*}=D_{1}\left(2-\sqrt{3}\right)\sim 0.28D_{1}$ is accordingly inferior to the diameter of $D_{3}\sim 0.4D_{1}$. One can therefore introduce smaller spheres across the cube face with only two optical contacts with the spheres with diameter $D_{3}=D_{1}-D_{2}$ and distance between four big spheres $D_{3}^{*}$. It is easily observable that the small spheres keep unstable under this packing configuration and can relax in four points of the free volume in contact with the big spheres.

It is however possible to reach a stable periodic construction if one implements into the packing configuration a rotation ellipsoid with the big and small axes as follows $\left(D_{3}^{*},D_{3}\right)=\left(D_{1}\left(2-\sqrt{3}\right),D_{1}\left(2-\sqrt{3}\right)\right)$, respectively. The total contact surface with fluids substantially increases to $S=S_{1}+S_{2}+S_{3}$ with three small spheres in any elemental cell, to become $S_{3}=3\pi ND_{3}^{2}=3\pi ND_{1}\left(7-4\sqrt{3}\right)$.

To enhance the decontamination rate, it is therefore desirable to use spheres with a diameter 5–10 times smaller than the distance between the large spherical ones and evaluate the total contact surface with contaminated fluids. If one subsequently packed “n” species of a smaller order into the free space in-between large spheres, an extended total contact area, $S_{tn}$, is secured with the contaminated fluid:

(1) $$S_{tn}=S_{1}+S_{2}+\cdots +S_{n}$$

When considering the analytical expression for each species of packing spheres, Equation (1) becomes reduced to a dependence on the diameter and volume of the total surface of every type of sphere. Here, $S_{1}=\pi D_{1}^{2}N$ and $S_{2}=\pi \left(\sqrt{3-1}\right)2D_{1}^{2}$ are the surfaces of the big and small spheres in the two lattices with the same number of nodes, N, in optical contact.

The number of spheres with the diameter $D_{i}$ was considered proportional to $N_{i}=V_{ft}/D_{i}^{3}$ which after the $N{\mathrm{th}}^{}$—packing is identical to that of the remanent free volume: $V_{fi}=V{\left(1-\rho \right)}^{i-1}$.

It is possible to continue filling the space in-between $D_{2}$—spheres with elements having a diameter *K* time less than $D_{2}$ ($D_{3}\sim 0.2–0.3\;\mathrm{mm}$). A relevant example is illustrated in Figure 4, where the space in-between large spheres (with a diameter $D_{1}\sim 2–3\;\mathrm{mm}$) is filled up with spheres with a diameter 10 times smaller (K=10 and $D_{2}\sim 0.2–0.3$ mm). If the space in between blue spheres (Figure 4a) are filled up with purple spheres, the situation is as that in Figure 4b. A new contact area is reached after every i-filling step of the order $S_{i}=4\pi V_{ft}/D_{i}$.

Accordingly, the free space becomes smaller than in the $\left(i+1\right){\mathrm{th}}^{}$ packing step. After the subsequent packing steps with spheres, the contact surface becomes:

(2) $$S_{i}=4\pi V{\left(1-\rho \right)}^{i-1}10^{n-1}/D_{i}$$

Equation (2) defines the sum of geometric series corresponding to the sphere in contact with the contaminated fluid, i.e., $V{\left(1-\rho \right)}^{i}$. The corresponding total area can be expressed as:

(3) $$S_{tn}=\frac{4\pi V\left(1-q^{n}\right)}{D_{1}\left(1-q\right)}$$

where $q=\left(1-\rho \right)K$. The number of spheres with diameter $D_{i}$ is proportional to $N_{i}=V_{ft}/D_{i}^{3}$. Equation (2) quantifies the major contribution of the metamaterial to the total surface (Equation (3)). This is obtained whenever the geometric progression ratio is superior to unity, q≫1. The main contribution to the total surface originates therefore from spheres with the smallest diameter, $D_{n}=10^{-\left(n-1\right)}D_{1}$. Accordingly, the total area of the composite metamaterial drastically increases when decreasing the diameter of the smallest packing fraction. The analyses were extended to the case when K>1 and $\left(1-\rho \right)<1$ to obtain an unity value of the geometrical progression ratio, $q=\left(1-\rho \right)K\sim 1$. The quartz spheres with a $D_{i}=D_{i-1}/K$ diameter hence bring a substantial contribution to the total area of composite metamaterial in contact with the contaminated fluid.

### 2.2. Optical Fibres

**Figure 5:**
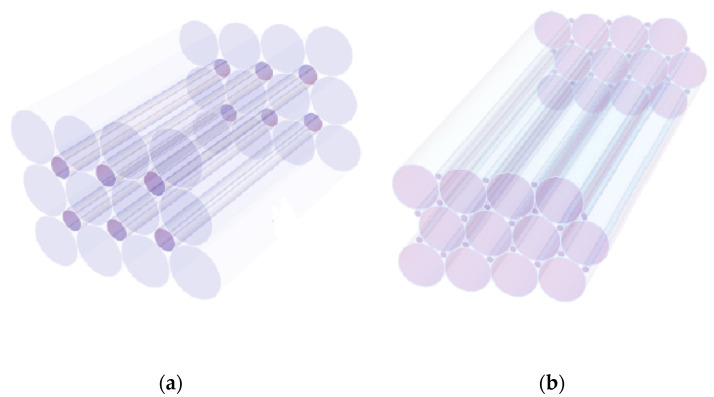
(**a**) Packing schemes with square; (**b**) hexagonal fibres fragments, respectively.

Similar calculations were carried out in the case of the packing procedure of optical fibres of identical lengths (*L*) but different diameters (*d_i_*) (Figure 5). It was shown that the square packing of fibres with a diameter $d_{1}$ allows the filing up of the remaining free space in between fibres with a smaller diameter, $d_{2}=d_{1}\left(\sqrt{2}-1\right)$, (Figure 5a). The entire fibres surface increases with the sum of the thick and thin fibres $S_{t}=S_{1}+S_{2}$. For any type of fibres, the total surface is equal to the product of the circular perimeter multiplied by the number of fragments and corresponding lengths: $S_{i}=\pi d_{i}LN_{i}$. The relation between the diameters $d_{1}$ and $d_{2}$ of the thick/thin fibres and the length of the square packing box, $L_{pb}$, is: $L_{pb}=N_{x}d_{1}$. One may assume that $\;N_{x}=\sqrt{N_{1}}$, with $N_{x}=N_{y}$ because the square box contains the same number of fibres along x or y directions, $N_{1}=N_{x}N_{y}$. It follows that the total surface of packed fibres of the same diameter is $S_{1}=\pi L_{pb}L\sqrt{N_{i}}$. According to Refs. [9,10,12], the total surface of the packed cylinders corresponding to unused space, during fluid decontamination is $S_{i}∼L_{pb}L\sqrt{N_{1}}$. The corresponding contact surface is $S_{2}=\pi L_{pb}L\sqrt{N_{2}}\left(\sqrt{2}-1\right)=S_{1}\left(\sqrt{2}-1\right)$. One obtains, accordingly, in the case of closed packing of fibres, an increase of total contact surface of the order of 44%.

The packing procedure can be extended to hexagonal arrangements of thick/thin fibres inside the decontamination core (Figure 5b). The fibres radius (r=d2) are in this case, r2 =r1 1−32, inferior to that in square packing.

The total surface becomes $S_{t}=S_{1}+S_{2}=2\pi r_{1}(1-\sqrt{3}/2)LN$. The decontamination volume is proportional to this surface multiplied by the penetration depth of UVC radiation. The decontamination volume is inferior to the free volume in between fibres because of the large distance among fibres with a diameter of (1–2) mm with respect to the UVC wavelength. This volume, *V_u_*, is defined by the surface of all fibres and the surface of the quartz tube, s, multiplied by the radiation penetration depth *k*. Thus, *V_u_* = (*A* + *s*)*L*pb. The free volume in between three fibres with a length of 100 cm and the diameter of 0.1 cm can be estimated from *V_f_* = *L*($\sqrt{3}$ − *π*/2)*r*^2^ = 0.16*r*^2^*L*.

The decontamination rate efficiency of the volume in between fibres inside the cylinder may therefore be estimated. Our approach resorts to the calculation of the effective volume in between the three fibres. The volume is equal to the length of the three fibres multiplied by the radiation penetration depth, κ∼λ/2, inside fluid: $V_{u}=\pi r\kappa L$. The contact efficiency of $E_{f}=V_{u}/V_{f}∼\lambda /r$ is therefore small for a wavelength within the range λ~250–280 nm.

The free volume, $V_{f}=V-V_{m}=\pi (R^{2}-Nr^{2})L$ is the difference between the total volume of the big cylinder $V=\pi R^{2}L$, with *R* = 2.5 cm *L* = 100 cm, and the volume of packed fibres of $V_{m}=\pi Nr^{2}L$.

## 3. Materials and Methods

Yeast fungi have been chosen as a relevant biological test agent due to their similarity and sometimes higher resistance to important pathogens.

Thus, according to [19,20,21], the yeast fungi resistance under UVC irradiation is close to that of Candida SP.

The resistance to prokaryote bacteria is also quite high. The application of the pro-posed method can therefore be safely exported to a large class of pathogens and does not necessitate dedicated experiments.

We used in experiments yeast colonies 1–30 µm in diameter.

**Figure 6:**
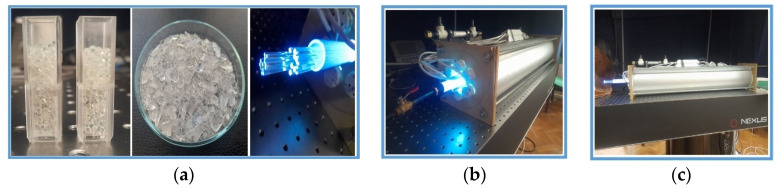
Decontamination core filled up with metamaterials. (**a**) Quartz spheres (**left**), crushed grains quartz (**center**) and fibre fragments (**right**); (**b**) The core submitted to irradiation; (**c**) Al cylinder for protection and loss minimization.

The fluids submitted to irradiation via metamaterials were yeast solutions (pseudohyphae or false hyphae) consisting of interconnected unicellular organisms [25,26,27,28]. UVC radiation with a pick on (240–260) nm, provided by either 6 Hg lamps or a Q-Smart 850 Q-Scan laser source, was directed to a tubular structure of 3.0 cm diameter and 90 cm length, filled up with either (1) (0.1–0.5) mm quartz spheres, (2) (2–5) mm crushed quartz particles or (3) 1 mm diameter optical fibre fragments (Figure 6a). The lamp’s decontamination core system was placed inside an aluminum cylinder of (15–20) cm diameter to protect the environment against UVC irradiation and minimize losses outside the cylinder (Figure 6b,c).

The yeast was fermented for 24 h before experiments. The stabilization of fungal colonies, in respect to their diameter and number, was observed during the whole period. This was reached for a mean number of fungal colonies, *n* = 750, with a diameter, *d*, of (4–12) µm [29]. The fibers were glued directly to the UVC side access.

The metamaterials used in the reported experiments exhibit a higher resistance to fluid circulation compared to the unpacked ones.

One should, however, mention that in the case of our closed system (of 0.9 m lengths), the amount of fluid under UVC radiation stays the same whether circulated once or several times. That is why the decontamination rate is several times more efficient when using packed metamaterials.

## 4. Results

**Figure 7:**
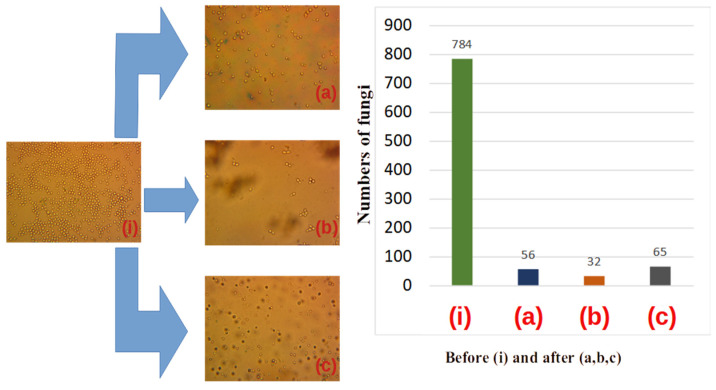
The decontamination rate of an earlier stage of preparation of the fungal solution (apr. after 15–20 min), see (**i**). (**a**) corresponds to repackaging spheres with diameters of 1–3 mm; (**b**) corresponds to crushed metamaterials with dimensions of approximately 0.01 mm–3 mm, and fig; (**c**) corresponds to repacking the core with spherical metamaterial of 1 mm–3 mm diameter. After 5 min of stationary decontamination, we obtain the histogram represented on the right-hand side of the figure. The initial colony count is 784. After 5 min of decontamination, 56 spherical metamaterials were observed, in crushed spheres 32 and in repackaged spheres 65.

**Figure 8:**
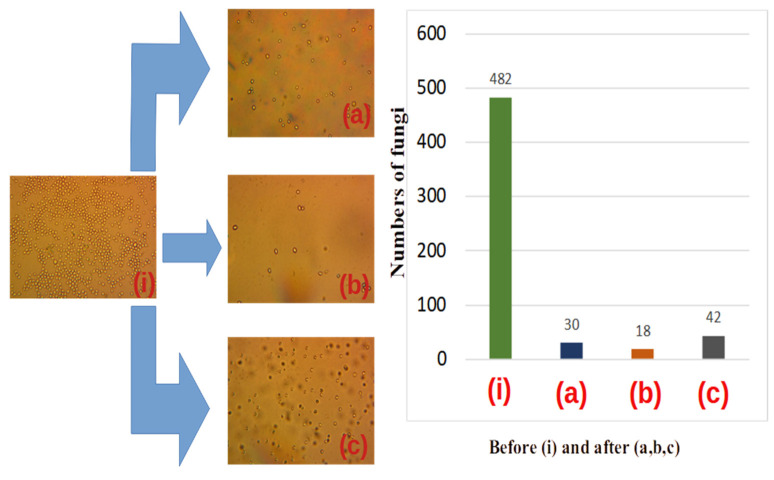
The decontamination rate after 24 h of fungal colony growth (**i**). (**a**) corresponds to repackaging spheres with diameters of 1–3 mm; (**b**) corresponds to crushed metamaterials with dimensions of approximately 0.01 mm–3 mm; (**c**) corresponds to repacking the core with spherical metamaterial of 1 mm–3 mm diameter. The histogram represented on the right side of the figure shows the decrease in the decontamination rate. The results were obtained in spherical metamaterials 30 colonies, in crushed spheres 18, and in repacked spheres 42.

Typical optical images of the initial yeast fungus colonies prior to decontamination are displayed in Figure 7 after 15–20 min of preparation of the solution: Figure 8 corresponds to the initial state of the developed fungal colonies after 24 h. The number of colonies per sample varied from 2 to 14. The size distribution seems to be aleatory, but the number of colonies decreases with dimension. More precisely, the connection between colony number, *n*, and diameter *Δ*, is given by Equation (4) [30].

(4) $$W\left(n,\Delta \right)=\frac{1}{\sqrt{2\pi \sigma_{n}^{2}}}exp\left[-\frac{{\left(n-n_{0}\right)}^{2}}{2\sigma_{n}^{2}}\right]\times \frac{1}{\sqrt{2\pi \sigma_{\Delta_{n}}^{2}}}exp\left[-\frac{{\left(\Delta_{n}-\Delta_{n_{0}}\right)}^{2}}{2\sigma_{\Delta_{n}}^{2}}\right]$$

Here, *W* stands for the Gaussian distribution, *n*_0_ is the initial number of colonies, while $\sigma_{\Delta_{n}}^{2}$ stands for size variation while $\sigma_{n}$ is the numerical variance of yeast colonies for the same diameter *Δ*. The average diameter and number of colonies in yeast solutions submitted to decontamination were experimentally determined, as in Ref. [30]. The values before decontamination are $n_{0}=9,$ $\sigma_{n}=2$ and $\sigma_{\Delta_{n}}=0.1/d_{sp},$ where $d_{sp}$ is the visualized diameter of the microscope image.

To investigate the role of the filling up of the free space between metamaterial elements in decontamination, three dedicated experiments were carried out using the setup in Figure 6. An increase in the decontamination rate is expected with the decrease of the free space in between metamaterial elements, in accordance with the predictions of the analyses in the previous section.

Quartz spheres of (1–2) mm (Figure 7), crushed quartz elements (Figure 8) and fibres of 1 mm diameter, were used to fill up the core tube and compare the performances of the contact surfaces. (magnification of 500×).

The decontamination stage is presented after 5 min of UVC irradiation (center) versus 1 day of fluid transit without irradiation (right) with respect to the initial status (left).

We observed that after 15–20 min the number of colonies was lower than after 24 h. I this sense, the size of the colonies has a large dispersion (diverse in diameter). As a rule, air bubbles prevailed in which the fungal colonies began to grow. After 24 h the number of colonies reached 800 in decreasing size dispersion. In the last case, the fungal colonies became more resistant to UVC radiation. In Figure 8, we represent the same experiments repeated after 24 h, the decontamination time being 5 min.

A significant result is, in our opinion, that after only 5 min of decontamination, a large part of yeast colonies was inactivated (see central parts of Figure 7 and Figure 8) for all used metamaterials: quartz spheres, crushed quartz elements, and optical fibers. Moreover, as visible from the figures, the mixture of crushed quartz elements with dimensions of (0.01–3) mm demonstrated the highest decontamination rate. This could be related to the smallest residual separation interval in this case compared to the quartz spheres and optical fibers, during the laminar flow of the contaminated fluid.

## 5. Discussion

The efficient decontamination under UVC irradiation opens new perspectives in the fabrication of innovative equipment with high productivity and tunability for the detection and elimination of bio/chemical contaminants.

Whenever good contact between the metamaterial elements (spheres, optical fibres and/or crushed elements) is reached, the light is “confined” and dispersed via evanescent waves in an extended volume of circulating fluids.

This objective, however, faces with two contradictory demands. Thus, one must allow for the free circulation of the fluid while the total contact surface should be concomitantly increased via reducing the dimensions of all metamaterial elements.

On the other hand, the contraction of the metamaterial dimensions’ elements causes the decrement of the flow speed due to higher friction. It also impedes the deep penetration of UVC radiation inside the metamaterial due to extended dispersion.

To surpass the apparent limits connected with fluids and irradiation, we propose the use of a combination of big and small metamaterials. Big elements will allow the radiation to penetrate the fluid, while small elements optimally disperse the radiation towards deeper regions of the metamaterial [31,32].

To reach this purpose, the packing of metamaterial elements (quartz spheres, crushed metamaterial, and optical fibres) is considered.

Depending on the respective metamaterial packing, the design of the decontamination core proves essential. Thus, taking into consideration the a priori known diameters of big and small spheres, one may obtain a superior repacked structure similar to the solid-state ones. Some defects in the repacking procedure may, however, persist, be observed as a significant modification, and can perhaps play an important role in the repacked structures. Test experiments of decontamination were carried out with packed element structures in the case of yeast, *Saccharomyces cerevisiae*, solutions because of the large resistance to UVC irradiation of this compound compared to microbes and viruses. We chose a mean value of the diameters of fungal colonies (of about 3–4 µm) and the mean value of the number of colonies at about 800.

We conducted a microanalysis investigation in two steps. One is the micro-biological analysis that is required to measure the size and number per volume of colonies in one drop of fungal solution crossing the UVC core, versus a drop that does not enter solution (see Figure 7 and Figure 8). A second investigation consisted of testing the multi-circulated products in comparison with samples that do not enter the core. The first experiment was carried out at a small time interval up to 5 min after the beginning of growing fungal colonies. The second one was performed after 24 h when the fungal colonies significantly increased and they began to interact with each other. One should mention here that in fact the spheres do overlap in this case with each other, and the atom–atom bond distance is less than the covalent radii of the bonded atom. The experiments have, however, different descriptions from a statistical point of view. The early (first) one contains fast studies of the development of fungal colonies. The distribution of colonies relative to their diameters in the early experiments contains a large dispersion compared to the later one (after 24 h). For example, in the first case one observed a relative dispersion of δ ≅ 0.3.

After 24 h, the dispersion becomes smaller (δ ≅ 0.15). The numbers of colonies also fluctuated in earlier experiments (an observed fluctuation relative number was δ ≅ 0.2). In experiments after 24 h, colonies practically come into contact with each other and the dispersion is very small δ ≅ 0.1.

These considerations support the experimental results displayed in Figure 7 and Figure 8, where a visual representation is also given in the form of histograms of the inactivation procedure after 5 min and one day, respectively.

An evident elimination of yeast colonies was demonstrated after UVC irradiation of quartz spheres, crushed metamaterial, and optical fibres mediated by multiple reflections via evanescent waves. The significant reduction of the yeast cell colonies number was obvious after 5 min only.

One notices a more efficient decontamination rate in the case of crushed metamaterials as compared to quartz spheres or optical fibres.

The yeast solution was less decontaminated in the presence of the metamaterials consisting of fibres and entire spheres. According to experimental evidence (Figure 7 and Figure 8), the decontamination is superior for a higher degree of packing in the case of given amounts of liquid and fungus colonies and exposure time of the liquid to UVC irradiation. In agreement with the experimental results, the best decontamination rate was reached using crushed metamaterials.

The yeast solution flows laminarly in between optical fibres so that a lot of fungus colonies keep out the UVC penetrated zone, which possibly stays at the origin of the smaller (unsatisfactory) decontamination rate in this case. These results serve, in our opinion, as a demonstration of the approach introduced in Section 2—“Packing of Metamaterial Elements”, according to which better packing ensures a superior decontamination rate due to the enhanced action of evanescent waves coming out of transparent objects crossed by UVC light.

A large increase in the decontamination rate is therefore expected with the optimum design and fabrication of repacking structures. Indeed, the decontamination rate is con trolled by the packing geometry, i.e., by the elements conducting to a better filling of the free space in between them.

## 6. Conclusions

An approach is proposed to substantially increase fluid decontamination efficiency under UVC irradiation by packing together large and small size metamaterials elements. The total surface of metamaterials in contact with contaminated fluids is, in this case, significantly increased, with beneficial effects upon decontamination. Multiple reflections of UVC radiation between denser packed metamaterial elements significantly improve the decontamination rate, mainly via evanescent waves.

A connection between optical element packaging, and the viability of the pathogen in the fluid was investigated and verified by test experiments with yeast solutions because of their superior resistance to UVC irradiation. Damage to yeast colonies anticipates, therefore, more consistent effects on relevant microbes and viruses. On this basis, a challenge can be opened for the design of UVC LED systems for efficient decontamination of fluids using optical resonances between metamaterial elements of different sizes. The decontamination of considerable, non-transparent flows of contaminated fluids can thus be ensured by inexpensive and easy-to-monitor technologies.

## Data Availability

Not applicable.
